# Functional analysis of the impact of *ORMDL3* expression on inflammation and activation of the unfolded protein response in human airway epithelial cells

**DOI:** 10.1186/1710-1492-9-4

**Published:** 2013-02-01

**Authors:** Karolynn J Hsu, Stuart E Turvey

**Affiliations:** 1 Division of Infectious and Immunological Diseases, Department of Pediatrics, BC Children’s Hospital and Child & Family Research Institute, University of British Columbia, Vancouver, BC, Canada

**Keywords:** Immune response, Epithelium, Cytokines, Chemokines, Host defense

## Abstract

**Background:**

The gene *ORMDL3* was shown to be associated with early-onset asthma susceptibility in multiple independent genome-wide and candidate-gene association studies. Asthmatic patients have elevated expression levels of this gene. *ORMDL3* encodes a transmembrane protein localized in the endoplasmic reticulum (ER) that may be involved in ER stress and inflammation. It is essential to validate the genetic associations linking *ORMDL3* with asthma through functional studies that confirm the biological relevance of this gene in disease. We investigated the effects of manipulating *ORMDL3* expression levels *in vitro* in airway cells on innate immune inflammatory responses, ER stress and activation of the unfolded protein response (UPR).

**Methods:**

*ORMDL3* expression levels were manipulated in airway cells using an overexpression plasmid and siRNA technologies. Successful modulation of ORMDL3 was confirmed at both the gene and protein level. The functional impact of modulation of *ORMDL3* expression levels on inflammatory responses and activation of the UPR were quantified using complementary cellular and molecular immunology techniques.

**Results:**

Cells with altered *ORMDL3* levels responded equally well to innate immune stimuli and produced similar levels of pro-inflammatory cytokines compared to wild-type cells. Treatment with ER stress inducers, thapsigargin and tunicamycin, resulted in activation of the unfolded protein response (UPR). However, we observed no difference in UPR activation in cells with *ORMDL3* knockdown compared to cells with normal *ORMDL3* levels.

**Conclusions:**

Our results suggest that *ORMDL3* variation in the airway epithelium is unlikely to play a significant role in modulating innate immune responses and the UPR in the lung.

## Introduction

Asthma and allergic diseases are rapidly becoming the most common chronic diseases in the developed world. Current asthma therapy treats symptoms of the disease, however it is ineffective in up to 25% of patients [1]. Asthma and allergic diseases are complex disorders caused by the interaction of various genetic and environmental factors [2-4].

Genome-wide association studies (GWAS) have been used to identify genes that may be involved in asthma pathogenesis [5]. Moffatt and colleagues first reported that multiple single nucleotide polymorphisms (SNPs) on chromosome 17q21 linked *ORMDL3* (orosomucoid 1-like 3) to the risk of developing childhood asthma [6]. This association has since been reproduced in multiple independent studies [7-14]. However, little work has been done to elucidate the biological and functional relevance of this gene in asthma. The disadvantage of these association studies is that they cannot differentiate between true causal SNPs and non-causal variants simply in linkage disequilibrium with disease-causing genes. It is therefore imperative to validate GWAS data through functional studies that confirm the biological relevance of a gene in disease.

SNP variants have also linked *ORMDL3* to inflammatory bowel disease (IBD) and Type I diabetes, suggesting that *ORMDL3* may be involved in dysregulation of the immune system [15,16]. Association of *ORMDL3* in both asthma and IBD is of interest because the lung and gut are composed of similar mucosal surface cells and these tissues are exposed to many potentially harmful antigens and allergens requiring tight regulation of the mucosal immune system [17]. This unique system is responsible for maintaining a delicate equilibrium between antigen responsiveness and tolerance and is therefore responsible for preventing hyper-reactivity [17]. Inappropriate immune responses to foreign components or commensal bacteria can lead to inflammation characteristic of asthma and IBD. Furthermore, the polymorphisms may be involved in regulation of mRNA expression of 17q21 locus genes, including *ORMDL3*[6]*.* The expression of *ORMDL3* was recently associated with elevated levels of IL-17 secretion [18] and *ORMDL3* was expressed at higher levels in the peripheral blood of patients with recurrent wheeze compared to controls [19]. This correlation further supports the hypothesis that *ORMDL3* is involved in immunity.

The *ORMDL3* gene is a member of a family of conserved endoplasmic reticulum (ER)-localized transmembrane proteins [20]. The functions of the *ORMDL* proteins are currently unknown, but a recent study suggested that ORMDL3 is involved in ER-mediated Ca^2+^ homeostasis and activation of the unfolded protein response (UPR) – ORMDL3 may inhibit sarco/endoplasmic reticulum Ca^2+^ ATPase (SERCA) activity [21,22]. Disruptions to ER Ca^2+^ concentrations can cause protein misfolding, and accumulation of these unfolded proteins can lead to ER stress [23,24]. UPR signaling cascades are initiated in response to this stress and have been shown to activate the JNK-AP-1 and NF-κB-IKK pathways [25-27]. The ER stress response and UPR, caused by changes in *ORMDL3* expression, can initiate inflammation through induction of cytokine production. This mechanism may explain the role of *ORMDL3* in asthma pathogenesis. Indeed, Miller *et al.* have shown that in mice *ORMDL3* is an allergen and cytokine (IL-4 or IL-13) inducible ER gene expressed predominantly in airway epithelial cells, and that it activates the ATF6 pathway of the ER localized UPR regulating expression of metalloprotease, chemokine, and oligoadenylate synthetase genes [28].

Although the symptoms of asthma are largely driven by dysregulated T helper type 2 (T_H_2) responses, innate immune responses are also involved in asthma pathogenesis [29,30]. Airway epithelia are central to host defense and immune regulation. These cells are among the first to encounter environmental insults and play an important role in shaping downstream immune responses. Any dysregulation of the innate immune response can result in hypersensitivity to environmental factors, leading to asthma symptoms.

Given the multiple lines of evidence suggesting that *ORMDL3* is involved in immunity, we investigated the role of the gene in innate immune responses of airway cells. We hypothesized that elevated *ORMDL3* levels result in heightened inflammatory responses that are associated with the asthmatic phenotype. Increased levels of ORMDL3 protein may in turn disrupt ER homeostasis, leading to ER overload and activation of the UPR, initiating inflammatory responses. Using an *in vitro* model, we manipulated *ORMDL3* expression in airway cells to determine whether a difference in basal *ORMDL3* expression affected inflammatory responses or activation of the UPR before and after stimulation.

## Materials and methods

### Cell culture

1HAEo^¯^ (1HAE) cells (SV40-transformed normal human airway epithelial cells) were cultured in DMEM-high glucose medium with 10% fetal calf serum (FCS), 2 mM L-glutamine, and 1 mM sodium pyruvate (HyClone). A549 cells (adenocarcinomic human alveolar basal epithelial cells) were cultured in F-12K medium supplemented with 10% fetal calf serum (FCS), 2 mM L-glutamine, and 1 mM sodium pyruvate (HyClone). Cells were incubated in a 37°C, 5% CO_2_ incubator. All cells were cultured under non-polarizing conditions.

### Cloning ORMDL3 cDNA into pEGFP-N1 vector

The *ORMDL3* gene was amplified from cDNA using forward primer 5^′^-CTAAGAATTCATGAATGTGGGCACAGCGCAC-3^′^ and reverse primer 5^′^-TACTGGTACCCCGTACTTATTGATTCCAAAAATCCGGACT-3^′^, introducing *Eco*RI and *Kpn*I restriction endonuclease sites, respectively. The *ORMDL3* PCR product was then inserted into a pEGFP-N1 eukaryotic expression vector (Clontech). *ORMDL3* and *eGFP* are in frame and produce a fusion protein with eGFP expressed at the C-terminus of ORMDL3. The construct was verified by sequencing and is denoted as pEGFP-ORMDL3. Protein is denoted as ORMDL3-eGFP.

### Cell transfection

A549 and 1HAE cell lines were transfected with pEGFP-ORMDL3, scramble (non-specific) or *ORMDL3*-specific siRNA (pre-designed by Qiagen) using Amaxa^®^ Cell Line Nucleofector^®^ Kit T (Lonza). Two *ORMDL3*-specific siRNAs were used. Concentrations used for transfection represent pooled siRNA concentration. Cells were seeded into a 24-well plate (BD Biosciences) at a density of 2x10^5^ cells/well for A549 cells or 1x10^5^ cells/well for 1HAE cells.

### Cell stimulation and immune response quantification

Twenty-four hours post-transfection, cells were stimulated with TNF-α (200 ng/ml) (eBioscience), *E. coli* K12 LPS (100 μg/ml) (InvivoGen), *S. typhimurium* flagellin (10-200 ng/ml) (InvivoGen), or IL-1β (200 ng/ml) (eBioscience). Stimulants and their concentrations were chosen based on published literature or past experiments [31-34]. Cells were stimulated for 24 hours. Supernatants were collected and analyzed for cytokine secretion. Pro-inflammatory cytokines, IL-6 and IL-8, were detected and quantified using Human IL-6 and IL-8 Ready-Set-Go!^®^ ELISA kits (eBioscience). Experiments were repeated three times (n = 3).

### ER stress induction and UPR activation

Cells were stimulated with tunicamycin (200 μg/mL) (Calbiochem) or thapsigargin (10 μM) (Sigma) for 2 or 4 hours to activate the UPR. For *ORMDL3* knockdown cells, stimulation was performed 24 hours post-transfection. RNA was extracted and expression of genes *XBP-1u, XBP-1s,* and *CHOP* were then quantified as markers of UPR activation. For measurement of p-eIF2α levels by Western blot, lysates from unstimulated cells with *ORMDL3* knockdown were collected 24 hours post-transfection. Experiments were repeated three times (n = 3).

### RNA isolation and reverse transcription

RNA was extracted from lysates using E.Z.N.A.^®^ Total RNA Kit (Omega Bio-Tek) according to the manufacturer’s protocol. Extracted RNA was reverse transcribed into cDNA using the SuperScript^®^ VILO™ cDNA Synthesis Kit (Invitrogen). Complement DNA was diluted to 200 ng/μl prior to quantification of gene expression by qPCR. This method was followed for all samples, unless otherwise stated (see PCR Array).

### Quantification of *ORMDL3* mRNA expression

Gene expression was calculated relative to *GAPDH* or *PPIA* (encoding cyclophilin A) and was quantified by SYBR Green chemistry (PerfeCTa™ qPCR SuperMix, Quanta Biosciences) using a 7300 Real Time PCR System (Applied Biosystems). Reactions were performed in triplicate using the following cycling conditions: 50°C for 2 mins, 95°C for 10 mins, [95°C for 15 s, 60°C for 1 min] x 40. The relative expression of the measured gene was calculated by the Pfaffl method [35]. The primers used are listed in Table 1.

**Table 1:** Quantitative PCR primer sequences

**Gene**	**NCBI accession**	**Forward (5**^′^** → 3**^′^**)**	**Reverse (5**^′^** → 3**^′^**)**	**CDS region**^**†**^	**Product size**
*GAPDH*	NM_002046.4	GCACCGTCAAGGCTGAGAACGG	CGACGTACTCAGCGCCAGCATC	c.173-286	114
*PPIA*	NM_021130	TAAAGCATACGGGTCCTGGCATCT	ATCCAACCACTCAGTCTTGGCAGT	c.269-369	101
*ACTB*	NM_001101.3	GTTGCGTTACACCCTTTCTT	ACCTTCACCGTTCCAGTTT	c.*16-*162	147
*DDIT3*	NM_001195053.1	GAAATGAAGAGGAAGAATCA	TTCTCCTTCATGCGCT	c.197-437	241
*XBP-1s*	NM_001079539.1	ATGGATGCCCTGGTTGCTGAAGA	TGCACCTGCTGCGGACTCA	c.415-504	90
*XBP-1u*	NM_005080.3	AGCACTCAGACTACGTGCACCTCT	CCAGAATGCCCAACAGGATATCAG	c.495-624	130
*ORMDL1*	NM_016467.4	AATGGCTGGTCCTTCAAGTGCT	ACCCTCACTGTGATGCCCTTTA	c.*121-*269	149
*ORMDL2*	NM_014182.4	ACACACTGGGAGCAAATGGACT	AGTGCGCAGCATCATACTTGGT	c.250-370	121
*ORMDL3*	NM_139280.2	TCAGGCAGCCAAAGCACTTTAACC	ACCCATCCCACACTTGCTTCCATA	c.*358-*496	139
*BCL6*	NM_001706.4	ACAATCCCAGAAGAGGCACGAAGT	GCTCGAAATGCAGGGCAATCTCAT	c.790-952	163
*CCL2*	NM_002982.3	TCGCTCAGCCAGATGCAATCAATG	TGGAATCCTGAACCCACTTCTGCT	c.65-259	195
*CCL5*	NM_002985.2	TGCCTGTTTCTGCTTGCTCTTGTC	TGTGGTAGAATCTGGGCCCTTCAA	c.*36-*127	92
*CSF2*	NM_000758.3	AAATGTTTGACCTCCAGGAGCCGA	GGTGATAATCTGGGTTGCACAGGA	c.185-357	173
*IL12A*	NM_000882.3	ATGATGGCCCTGTGCCTTAGTAGT	AGGGCCTGCATCAGCTCATCAATA	c.457-611	155
*IL13RA1*	NM_001560.2	GTCCCAGTGTAGCACCAATGA	CAGTCACAGCAGACTCAGGAT	c.297-391	95
*ADRB2*	NM_000024.5	TCATCATGGGCACTTTCACCCTCT	AGCTCCTGGAAGGCAATCCTGAAA	c.830-1016	187
*VEGFA*	NM_001025366.2	TTCAGGACATTGCTGTGCTTTGGG	TGGGCTGCTTCTTCCAACAATGTG	c.*778-*969	192
*IL23A*	NM_016584.2	TCGGTGAACAACTGAGGGAACCAA	TGGAATCTCTGCCCACTTCCACTT	c.-140- -54	87

^†^ c.-number if in the 5^′^UTR, c.*number if in the 3^′^UTR.

### Western blot analysis

Cells were lysed in 50 μl RIPA Buffer + 1x HALT™ protease inhibitor (Thermo Scientific). Cell debris were removed by centrifugation: 18,000 x *g* for 10 min at 4°C. Proteins were analyzed by standard Western blotting protocols where they were transferred onto Immobilon^®^-FL transfer membrane (Millipore). Antibodies used for Western blot analysis were: monoclonal anti-GFP antibody 1:10,000 (Clontech), anti-ACTB antibody 1:6,000 (Cell Signaling), anti-p-eIF2α 1:500 (Cell Signaling) and IRDye^®^ 680 or 800 secondary antibodies 1:8000 (Li-cor). Western blots were visualized using an Odyssey Infrared Imaging System (Li-cor).

### PCR array

1HAE cells co-transfected with pEGFP-ORMDL3 and *ORMDL3* siRNA (low *ORMDL3* expression) were compared to cells co-transfected with pEGFP-ORMDL3 and scramble siRNA (high *ORMDL3* expression) at two time-points (2 and 24 hours) after TNF-α stimulation. Extracted RNA was reverse transcribed into first strand cDNA using the RT^2^ First Strand Kit (SABiosciences, Qiagen). Protocol as described by the manufacturer was followed.

Two RT^2^ Profiler PCR arrays (SABiosciences, Qiagen), profiling expression of 84 genes each, were used: Human Cytokines & Chemokines and Allergy & Asthma (see Additional file 1 for complete list). Complementary DNA template was mixed with RT^2^ SYBR^®^ Green qPCR Mastermix (SABiosciences, Qiagen) as follows: 1350 μL SYBR Green Master Mix, 1248 μL nuclease-free H_2_O, and 102 μL cDNA (~200 ng/μL). Note: these volumes were used as recommended by the manufacturer for use with a 7300 Real Time PCR System (Applied Biosystems). Template was then aliquoted into PCR plates containing pre-dispensed primers. Cycler program as provided by the manufacturer was used. Results were analyzed using the PCR Array Data Analysis Web Portal.

### Statistical analysis

Data are shown as mean ± SEM of three separate experiments. Results were analyzed using one-way ANOVA with Bonferroni post-test. Statistical analysis was performed using GraphPad Prism5 (GraphPad Software, Inc.). Differences with p < 0.05 were considered significant.

## Results

### *ORMDL3* modulation in airway cells

In order to determine functional impact of *ORMDL3* modulation, knockdown of the gene was performed using siRNAs. A549 and 1HAE cells were transfected with scramble (non-specific) or *ORMDL3-*specific siRNA. Modeling variation expected to occur in the human population, we achieved 40-70% knockdown of *ORMDL3* gene expression using siRNA concentrations of 50 nM-500 nM. We also confirmed that *ORMDL3-*specific siRNA did not affect expression of genes in the same family, *ORMDL1* or *ORMDL2* (Additional file 2). Sequences of primers used for qPCR are listed in Table 1.

ORMDL3 has 84% and 80% protein sequence homology to ORMDL1 and ORMDL2, respectively. This presented a challenge for confirming knockdown of ORMDL3 protein because commercially available antibodies detect all three proteins. Therefore, we were unable to show ORMDL3 protein knockdown in cells transfected with siRNA alone. To overcome this limitation, we constructed a fusion protein, where ORMDL3 is tagged with an eGFP protein. Co-transfection of this overexpression plasmid with *ORMDL3*-specific siRNA enabled us to knockdown *ORMDL3* and detect changes at the protein level using an anti-GFP antibody.

**Figure 1:**
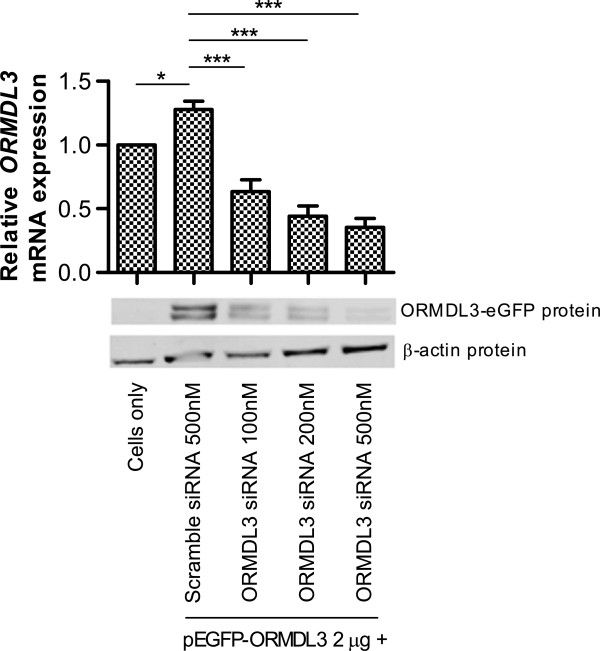
**Experimental manipulation of *ORMDL3 *expression.** Graph (top) shows relative *ORMDL3* transcript levels. Data represents the mean ± SEM of three experimental repeats. Statistical analysis was performed using one-way ANOVA with Bonferroni post-test. * p < 0.05, *** p < 0.001. Western blot analysis (bottom) shows protein expression of ORMDL3-eGFP and β-actin as a loading control. Blot is representative of three independent experiments.

Airway cells were co-transfected with both pEGFP-ORMDL3 and siRNA (scramble or *ORMDL3*). Protein and mRNA were analyzed for gene knockdown. At the transcript level, we observed (Figure 1) a small but significant increase (p < 0.05) in *ORMDL3* expression in the cells transfected with pEGFP-ORMDL3 and scramble siRNA compared to cells alone, confirming successful overexpression of the *ORMDL3* gene. Transfection with pEGFP-ORMDL3 and increasing concentrations of *ORMDL3* siRNA resulted in a titration effect of increasing *ORMDL3* knockdown. One advantage to constructing a fusion protein, ORMDL3-eGFP, is that eGFP is only expressed with expression of ORMDL3. Therefore, when cells are co-transfected with pEGFP-ORMDL3 and siRNA, knockdown of ORMDL3 protein could subsequently be detected by immunoblot analysis using the anti-GFP antibody. Expression of the ORMDL3-eGFP protein was confirmed by Western blot of whole cell lysate collected from the cells in each condition. Figure 1 shows knockdown of ORMDL3-eGFP protein, confirming that *ORMDL3* siRNA affects protein expression. The double band may be explained by either variation in mRNA splicing or post-translational modifications to the fusion protein, such as acetylation, methylation, myristylation, phosphorylation, or glycosylation.

### *ORMDL3* knockdown does not affect IL-6 or IL-8 production following innate immune activation

**Figure 2:**
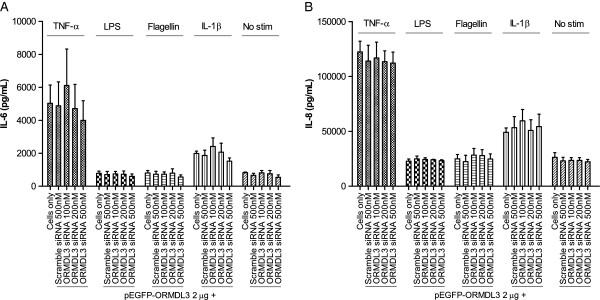
**Cytokine production in cells with ORMDL3 knockdown. A**) Secreted IL-6 and **B**) IL-8 levels after cell stimulation. 1HAE cells were stimulated with TNF-α, LPS, flagellin, or IL-1β for 24 hours. Data represent the mean ± SEM of three experimental repeats. Statistical analysis was performed using one-way ANOVA with Bonferroni post-test.

1HAE cells transfected with pEGFP-ORMDL3 and scramble or *ORMDL3-*specific siRNA were stimulated 24 hours post-transfection and supernatants were collected 48 hours post-transfection. Stimuli used were TNF-α, IL-1β, LPS, and flagellin. TNF-α and IL-1β were chosen because both are early response cytokines that perpetuate acute inflammatory processes. LPS and flagellin, in contrast, are common microbial antigens recognized by the innate immune system. Two classic and biologically-relevant NF-κB-induced cytokines with important roles in innate immunity, interleukin-6 (IL-6) and interleukin-8 (IL-8), were measured by ELISA. Despite confirmation of *ORMDL3* mRNA and protein knockdown, we did not observe any impact on IL-6 or IL-8 production after stimulation as shown in Figure 2A-B. Although the cells have low baseline responsiveness to LPS and flagellin, our results indicate that *ORMDL3* knockdown does not enhance sensitivity to these stimuli. Similar results were obtained in A549 cells, as well as 1HAE cells transfected with siRNA alone (Additional file 2).

### *ORMDL3* knockdown does not enhance UPR activation upon stimulation

We next explored the effects of *ORMDL3* expression on activation of the UPR. Initiation of the UPR is mediated by one or more of the ER-membrane protein sensors: PKR-like eukaryotic initiation factor 2α kinase (PERK), inositol requiring enzyme 1 (IRE1), and activating transcription factor-6 (ATF6) [23]*.* Activation of any of the three pathways initiates signaling cascades that mediate changes to relieve ER stress. The gene *XBP-1* is a substrate for IRE1 ribonuclease [24]. Upon activation of the IRE1 pathway, the IRE1 ribonuclease removes a 26-bp intron from the unspliced variant, *XBP-1u,* which results in the spliced variant, *XBP-1s*[36]*.* This spliced variant is the active form of the gene that contributes to ER stress responses. *CHOP* transcription, in contrast, can be induced by the PERK and ATF6 pathways [24]. Phosphorylated eIF2α (p-eIF2α) is an early marker of PERK pathway activation and is upstream of *CHOP* induction [23]. Expression changes in these three genes that signify UPR activation, *XBP-1u, XBP-1s,* and *CHOP*, were determined by qPCR. We also evaluated phosphorylation of eukaryotic initiation factor 2α (eIF2α) by Western blot, as modulation of *ORMDL3* expression has been reported to influence eIF2α phosphorylation [21].

**Figure 3:**
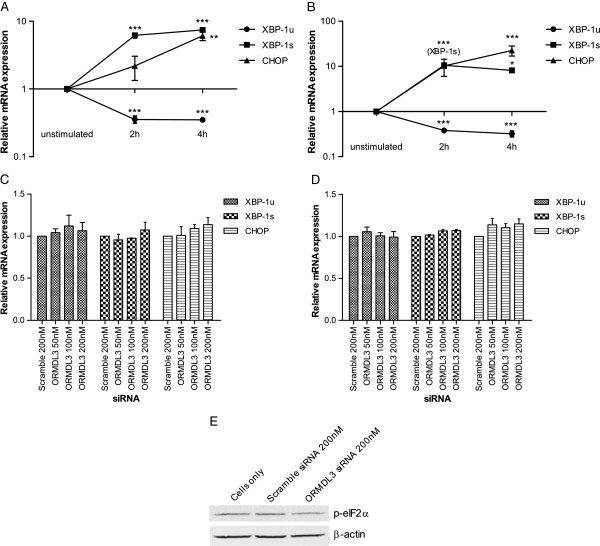
**Unfolded protein response activation in cells with *ORMDL3 *knockdown.** ER stress was induced in 1HAE cells by stimulation with **A**) tunicamycin or **B**) thapsigargin for 2 or 4 hours. Relative expression levels of *XBP-1u*, *XBP-1s*, and *CHOP* were quantified and compared to unstimulated cells. 1HAE cells with *ORMDL3* knockdown were stimulated with **C**) tunicamycin or **D**) thapsigargin for 4 hours. Relative gene expression levels were quantified and compared to cells transfected with scramble (a non-specific) siRNA. Data represent the mean ± SEM of three experimental repeats. Statistical analysis was performed using one-way ANOVA with Bonferroni post-test. *** p < 0.001, ** p < 0.01, * p < 0.05. **E**) Western blot analysis of p-eIF2α with β-actin loading control. Lysates are from unstimulated 1HAE cells 24 hours after *ORMDL3* knockdown. Blot is representative of three independent experiments.

As positive controls for UPR activation, we stimulated 1HAE cells with tunicamycin and thapsigargin. Both are inducers of ER stress – tunicamycin inhibits N-linked glycosylation and thapsigargin inhibits the SERCA pump causing ER calcium stores to be depleted [37]. Quantitative measurement of transcript levels showed that both *XBP-1s* and *CHOP* increased, while *XBP-1u* decreased upon stimulation with either tunicamycin or thapsigargin (Figure 3A-B). This confirmed the utility of measuring these genes to monitor UPR activation.

At baseline (unstimulated cells), variation in *ORMDL3* expression did not induce UPR activation in A549 or 1HAE cells (Additional file 2). 1HAE cells with *ORMDL3* knockdown were also stimulated with tunicamycin or thapsigargin (Figure 3C-D). In both conditions, *ORMDL3* knockdown did not show increased UPR activation compared to the negative control. Furthermore, levels of phosphorylated-eIF2α were indistinguishable between *ORMDL3* knockdown cells and controls (Figure 3E).

### Impact of *ORMDL3* knockdown on the expression of multiple genes involved in inflammation, asthma & allergy

To expand our search for immune functions potentially altered by *ORMDL3*, the expression of 168 genes was determined at two time points (2 and 24 hours) following stimulation with TNF-α. We performed PCR arrays to profile expression of cytokines, chemokines, and key genes involved in asthma and allergy (a complete list of genes that were studied can be found in Additional file 1). 

**Figure 4:**
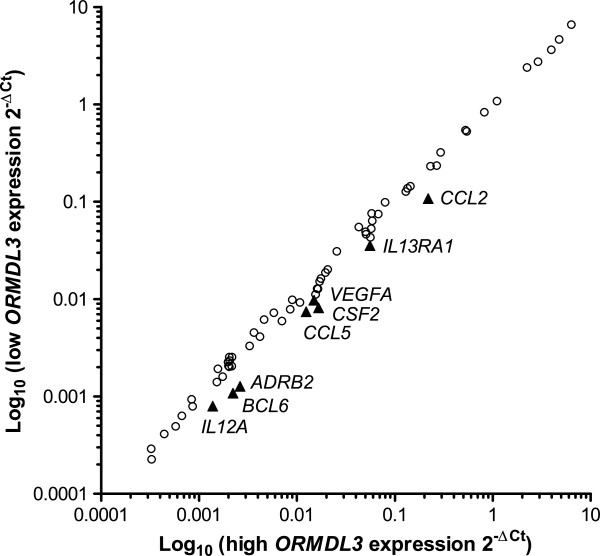
**PCR array.** Results shown are 168 genes profiled from two PCR arrays: Allergy & Asthma and Human Cytokines & Chemokines (Qiagen). 1HAE cells with high or low *ORMDL3* expression were stimulated with TNF-α for 24 hours and differences in gene expression were compared. Comparing low *ORMDL3* expression to high *ORMDL3* expression, circles are genes with less than 1.5 fold change and triangles are genes down-regulated by more than 1.5 fold.

Gene expression was compared between 1HAE cells with high (plasmid + 500 nM scramble siRNA) and low (plasmid + 500 nM of *ORMDL3* siRNA) *ORMDL3* expression that were stimulated for 24 hours with TNF-α. Stimulation with TNF-α induced a robust inflammatory response in the cells, enabling us to observe whether variation in basal *ORMDL3* levels impacts the immune response. These arrays identified eight genes (*CCL2, TSLP, CSF2, CCL5, VEGFA, ADRB2, IL1RL1,* and *IL13RA1*), shown in Figure 4, that appeared to be differentially regulated by more than 1.5 fold and that were expressed at relatively high levels (average threshold cycle <30). However, upon replication to validate these results, we determined that the differences were not statistically significant.

The same arrays were performed on cells stimulated for 2 hours with TNF-α. The same conditions for “high” and “low” *ORMDL3* expression were used. From our results, we identified only one gene, *IL23A*, that was differentially regulated by more than 1.5 fold (fold regulation −2.37) and amplified at cycle <30. Replication and comparison of *IL23A* expression between knockdown conditions yielded no significant difference. No other genes, as identified in the previous arrays, were found to be differentially regulated at this time point. These results suggest that *ORMDL3* variation does not have a meaningful impact on expression of a large panel of immune-related genes in airway epithelial cells.

## Discussion

Asthma is a complex disease affecting many individuals in the developed world. Genome-wide association studies have recently been used to identify genetic causes for such complex diseases. One particular gene, *ORMDL3,* is of interest because of its association with asthma, IBD, and Type I diabetes – all of which are caused by immune-mediated pathology [6,10,22,38,39]. The gene *ORMDL3* is an ER-membrane protein and is potentially involved in Ca^2+^-signaling in the ER and sphingolipid synthesis [20,21,40]. It has also been correlated to activation of the UPR, though the mechanisms remain unclear [21]. Activation of the UPR may be biologically relevant, as ER stress, the UPR, and inflammation have all been linked [23]. However, the functional role *ORMDL3* in the pathogenesis of asthma has yet to be elucidated.

Airway epithelial cells play an important role in innate immunity and in the development of asthma. Current findings in literature indicate that *ORMDL3* is involved in immunity and that asthmatics have higher expression of the gene than non-asthmatics [18,21,22]. A recent study by Miller *et al.* also investigated the role of *ORMDL3* in airway epithelial cells. They reported that *in vitro* overexpression of *ORMDL3* activated the ATF6 pathway of the UPR and induced expression of several genes with potential importance in the pathogenesis of asthma [28]. Our investigation, in contrast, focuses on the effect of variation of *ORMDL3* expression levels, at baseline, on the innate immune responsiveness of airway epithelial cells. By manipulating *ORMDL3* expression *in vitro* to mimic differences in gene expression established between asthmatics and healthy individuals, we aimed to understand the role of *ORMDL3* on the innate immune response and UPR activation status in airway epithelial cells. This method ensured control and the confidence that any effect on the innate immune response was in fact correlated with a change in *ORMDL3* expression levels. If the same experiments were performed on *ex vivo* airway cells of patients, genetic and other differences between individuals could have affected the results.

After knockdown of *ORMDL3 in vitro,* cells were stimulated with cytokines (TNF-α, IL-1β) or common microbial components (LPS, flagellin). We monitored production of interleukin-6 (IL-6) and interleukin-8 (IL-8) (alias CXCL8), two pro-inflammatory cytokines produced by airway cells that are relevant in asthma pathogenesis. Specifically, IL-6 is elevated in individuals with asthma [41] and is also regulated by ATF6 during activation of the UPR [42]. Similarly, transfection of ORMDL3 into human airway epithelial cells triggers ATF6 activation and IL-8 secretion [28]. However, in our experimental system, variation in *ORMDL3* expression levels did not affect NF-κB-induced innate immune production of IL-6 and IL-8 in airway epithelial cells.

We next explored the effects of *ORMDL3* expression on activation of the UPR. UPR signaling cascades are initiated in response to ER stress, and restoration of homeostasis is achieved by attenuating translation, restoring protein folding, or degrading misfolded proteins [24]. Although often associated with abnormal physiological conditions, the UPR plays a central beneficial role in normal physiology; as illustrated by the role of the UPR in terminal B cell differentiation which requires a massive increase in the biosynthetic capacity to synthesize antibodies in response to infection [43]. However, the ER stress response and UPR can also initiate inflammation through induction of cytokine production or activation of transcriptional regulators of inflammatory genes. Cytokines IL-6 and IL-8 are examples of genes that may be induced by UPR activation [23]. ER stress and the UPR have been implicated in many immune-related diseases including IBD, diabetes, chronic obstructive pulmonary disease (COPD), arthritis, and neurodegenerative inflammatory diseases [44]. It is poorly understood whether ER stress is an underlying cause of disease or if its induction is a result of chronic inflammation. Indeed, it is possible that environment factors such as infection or inhalation of smoke particles can activate the UPR, triggering the onset of lung disease in genetically predisposed individuals [45]. However, it is also possible that ER stress is exacerbated by inflammation and contributes to the perpetuation of the disease.

Cantero-Recasens *et al.* previously reported that *ORMDL3* overexpression activated the PERK pathway, but did not affect the IRE1 pathway of the UPR [21]. In contrast, Miller *et al.* reported that *ORMDL3* overexpression activated the ATF6 pathway, but not the PERK or IRE1 pathways [28]. In our study, we chose four markers of UPR activation: *XBP-1u, XBP-1s*, *CHOP*, and p-eIF2α. With activation of the UPR, we expect downregulation of *XBP-1u* and upregulation of *XBP-1s* and *CHOP.* However, our results demonstrate that knockdown of *ORMDL3* does not activate the UPR, in either unstimulated or stimulated cells. Immunoblot analysis also showed no change in p-eIF2α levels with *ORMDL3* knockdown. Furthermore, downstream markers of UPR activation, IL-6 and IL-8 cytokines, were produced at similar levels in unstimulated cells with varying *ORMDL3* levels. This further supports our results that *ORMDL3* does not activate the UPR. Differences in our results compared to previous work might be due to the different types of cells, conditions, or markers used. It is possible that the effects of variation in *ORMDL3* expression are a cell type-dependent phenomenon. While no effect on the inflammatory response was detected in airway cells, other cells types such as dendritic cells or T cells may be affected by altered *ORMDL3* expression. Observations made by Lluis *et al.* suggest that the 17q21 locus may potentially play a role in T-cell development [18].

Taking a broader approach, PCR arrays looking at expression of 168 common immunity genes were performed. We reasoned that although *ORMDL3* levels may not affect the production of IL-6 or IL-8 cytokines, perhaps they were impacting gene expression of other important immune genes, such as *IL-33*, *IL-25* and *TSLP*, which have all been implicated in asthma pathogenesis [46]. Verification of differential expression of these genes at a transcript level, however, did not show any significant changes between the *ORMDL3* knockdown conditions. This suggests that altering *ORMDL3* expression does not have a profound effect on the expression of innate immune genes upon stimulation in the airway epithelia. However, there may be other genes that are affected that were not investigated in this study. Pfeifer *et al.* recently showed that IL-17C cytokine is expressed by human bronchial epithelial cells and is induced by bacterial infection [47]. It may be worthwhile in future experiments to investigate a broader range of immune-related genes. Interestingly, we did not observe changes to expression of the genes reported by Miller *et al., MMP-9, CCL-20, CXCL-10, CXCL-11*, or *IL-8.* This variance may be explained by differences in experimental conditions. Our study examined outcomes in gene expression after stimulation of cells co-transfected with *ORMDL3* and *ORMDL3-*specific siRNA, while the other study used a different experimental approach.

Although this study focused exclusively on the potential role of *ORMDL3* in asthma pathogenesis, it is possible that neighboring genes such as *GSDML* contribute to disease susceptibility at this locus. Many groups consider *ORMDL3* as an ‘asthma gene’; however, it should be acknowledged that the identified SNPs associating this gene to asthma susceptibility are not located in the gene itself. Even so, these polymorphisms have been consistently correlated with increased odds of asthma risk, highlighting the importance of this locus in disease susceptibility [6-11,13,14].

Our data show that variation in *ORMDL3* expression is not correlated with differential innate immune responses to stimuli or activation of the UPR *in vitro* in airway epithelial cells. Taken together, our results are biologically relevant because they suggest that normal human variation of *ORMDL3* expression is not likely an important factor in increasing the innate immune response of airway cells we observe in asthmatics. Despite these results, this gene remains an important candidate for asthma susceptibility. More research is required to elucidate its role in asthma pathogenesis and its potential role as an initial trigger of inflammation. By increasing our understanding of the mechanisms responsible for allergic and atopic disease development, new treatments can then be developed. Thus, we can reduce inflammatory responses by targeting the potential triggers, rather than the symptoms, of the disease. In doing so, we will ultimately reduce the morbidity, mortality, and socio-economic burden of asthma and related allergic diseases.

## Competing interests

The authors declare that they have no competing interests.

## Authors’ contributions

KJH performed the research. All authors designed the research, analyzed the data, and drafted the manuscript. Both authors read and approved the final manuscript.

## Supplementary Material

Additional file 1 Genes analyzed by PCR array. Click here for file

Additional file 2 Additional figures. Click here for file
