# Supplementary material for: Functional analysis of the impact of ORMDL3 expression on inflammation and activation of the unfolded protein response in human airway epithelial cells
Source: Allergy Asthma Clin Immunol. 2013 Feb 1;9(1):4. doi: 10.1186/1710-1492-9-4 (PMC3651386; doi:10.1186/1710-1492-9-4)
Supplement: Additional file 1 — Genes analyzed by PCR array. [file 1710-1492-9-4-S1.pdf]

Table 1. Genes analyzed by PCR array

Human Cytokines & Chemokines Array		Allergy & Asthma Array	
**Gene**	**NCBI accession**	**Gene**	**NCBI accession**
ADIPOQ	NM_004797	*ADAM33*	NM_025220
BMP2	NM_001200	*ADRB2*	NM_000024
BMP4	NM_130851	*ALOX5*	NM_000698
BMP6	NM_001718	*AREG*	NM_001657
BMP7	NM_001719	*ARG1*	NM_000045
C5	NM_001735	*BCL6*	NM_001706
CCL1	NM_002981	*CCL11*	NM_002986
CCL11	NM_002986	*CCL17*	NM_002987
CCL13	NM_005408	*CCL2*	NM_002982
CCL17	NM_002987	*CCL22*	NM_002990
CCL18	NM_002988	*CCL24*	NM_002991
CCL19	NM_006274	*CCL26*	NM_006072
CCL2	NM_002982	*CCL5*	NM_002985
CCL20	NM_004591	*CCL8*	NM_005623
CCL21	NM_002989	*CCR3*	NM_001837
CCL22	NM_002990	*CCR4*	NM_005508
CCL24	NM_002991	*CCR8*	NM_005201
CCL3	NM_002983	*CD40LG*	NM_000074
CCL5	NM_002985	*CHI3L1*	NM_001276
CCL7	NM_006273	*CHIA*	NM_201653
CCL8	NM_005623	*CLC*	NM_001828
CD40LG	NM_000074	*CLCA1*	NM_001285
CNTF	NM_000614	*CMA1*	NM_001836
CSF1	NM_000757	*CPA3*	NM_001870
CSF2	NM_000758	*CRLF2*	NM_001012288
CSF3	NM_000759	*CSF2*	NM_000758
CX3CL1	NM_002996	*CSF3R*	NM_000760
CXCL1	NM_001511	*CYSLTR1*	NM_006639
CXCL10	NM_001565	*EPX*	NM_000502
CXCL11	NM_005409	*FCER1A*	NM_002001
CXCL12	NM_000609	*FOXP3*	NM_014009
CXCL13	NM_006419	*GATA3*	NM_002051
CXCL16	NM_022059	*PTGDR2*	NM_004778
CXCL2	NM_002089	*ICOS*	NM_012092
CXCL5	NM_002994	*IFNG*	NM_000619
CXCL9	NM_002416	*IFNGR2*	NM_005534
FASLG	NM_000639	*IL10*	NM_000572
GPI	NM_000175	*IL12A*	NM_000882

IFNA2	NM_000605	*IL12B*	NM_002187
IFNG	NM_000619	*IL13*	NM_002188
IL10	NM_000572	*IL13RA1*	NM_001560
IL11	NM_000641	*IL13RA2*	NM_000640
IL12A	NM_000882	*IL17A*	NM_002190
IL12B	NM_002187	*IL17RB*	NM_018725
IL13	NM_002188	*IL18*	NM_001562
IL15	NM_000585	*IL1RL1*	NM_016232
IL16	NM_004513	*IL21*	NM_021803
IL17A	NM_002190	*IL25*	NM_022789
IL17F	NM_052872	*IL2RA*	NM_000417
IL18	NM_001562	*IL3*	NM_000588
IL1A	NM_000575	*IL31*	NM_001014336
IL1B	NM_000576	*IL33*	NM_033439
IL1RN	NM_000577	*IL3RA*	NM_002183
IL2	NM_000586	*IL4*	NM_000589
IL21	NM_021803	*IL4R*	NM_000418
IL22	NM_020525	*IL5*	NM_000879
IL23A	NM_016584	*IL5RA*	NM_000564
IL24	NM_006850	*IL9*	NM_000590
IL27	NM_145659	*KIT*	NM_000222
IL3	NM_000588	*KITLG*	NM_003994
IL4	NM_000589	*LTB4R*	NM_181657
IL5	NM_000879	*MAF*	NM_005360
IL6	NM_000600	*MMP9*	NM_004994
IL7	NM_000880	*MRC1*	NM_002438
IL8	NM_000584	*MS4A2*	NM_000139
IL9	NM_000590	*PDCD1*	NM_005018
LIF	NM_002309	*PMCH*	NM_002674
LTA	NM_000595	*POSTN*	NM_006475
LTB	NM_002341	*PPARG*	NM_015869
MIF	NM_002415	*PRG2*	NM_002728
MSTN	NM_005259	*RETNLB*	NM_032579
NODAL	NM_018055	*RNASE2*	NM_002934
OSM	NM_020530	*RNASE3*	NM_002935
PPBP	NM_002704	*RORC*	NM_005060
SPP1	NM_000582	*SATB1*	NM_002971
TGFB2	NM_003238	*SIGLEC8*	NM_014442
THPO	NM_000460	*STAT5A*	NM_003152
TNF	NM_000594	*STAT6*	NM_003153
TNFRSF11B	NM_002546	*TBX21*	NM_013351

TNFSF10	NM_003810	*TGFB1*	NM_000660
TNFSF11	NM_003701	*TNFRSF4*	NM_003327
TNFSF13B	NM_006573	*TNFSF4*	NM_003326
VEGFA	NM_003376	*TPSAB1*	NM_003294
XCL1	NM_002995	*TSLP*	NM_033035
