# Supplementary material for: Functional analysis of the impact of ORMDL3 expression on inflammation and activation of the unfolded protein response in human airway epithelial cells
Source: Allergy Asthma Clin Immunol. 2013 Feb 1;9(1):4. doi: 10.1186/1710-1492-9-4 (PMC3651386; doi:10.1186/1710-1492-9-4)
Supplement: Additional file 2 — Additional figures. [file 1710-1492-9-4-S2.pdf]

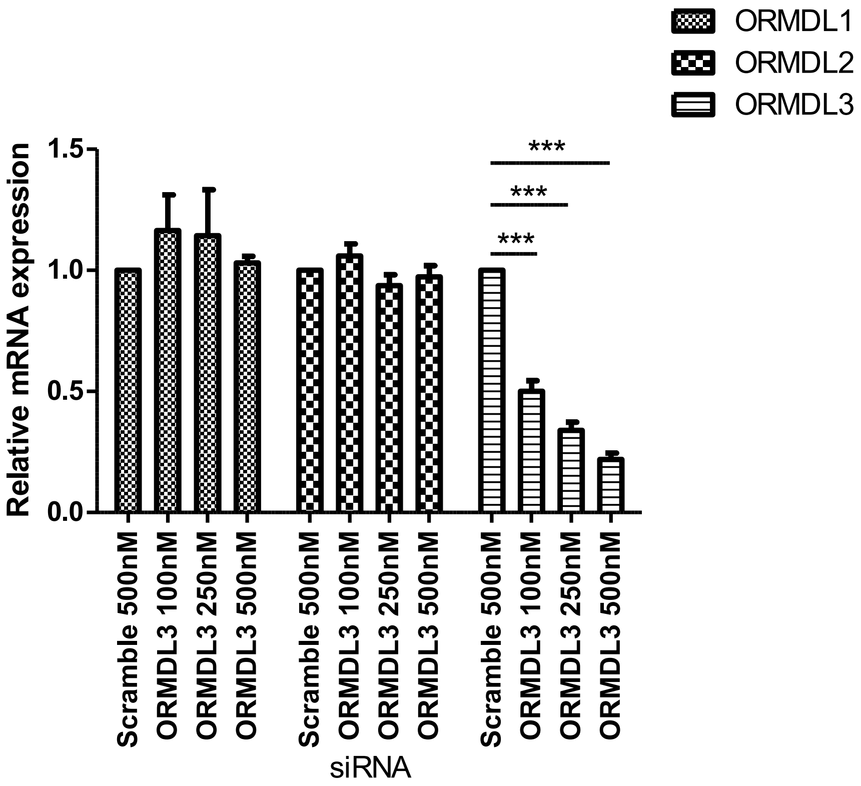

Specificity of *ORMDL3* siRNA. Relative expression levels of *ORMDL1, ORMDL2,* and *ORMDL3* were quantified 72 hours after transfection with *ORMDL3* siRNA in A549 cells. Gene expression is compared relative to cells transfected with scramble siRNA, a non-specific siRNA (negative control). Data represent the mean ± SEM of three experimental repeats. Statistical analysis was performed using one-way ANOVA with Bonferroni post-test. *** signifies $p < 0.001$.

A

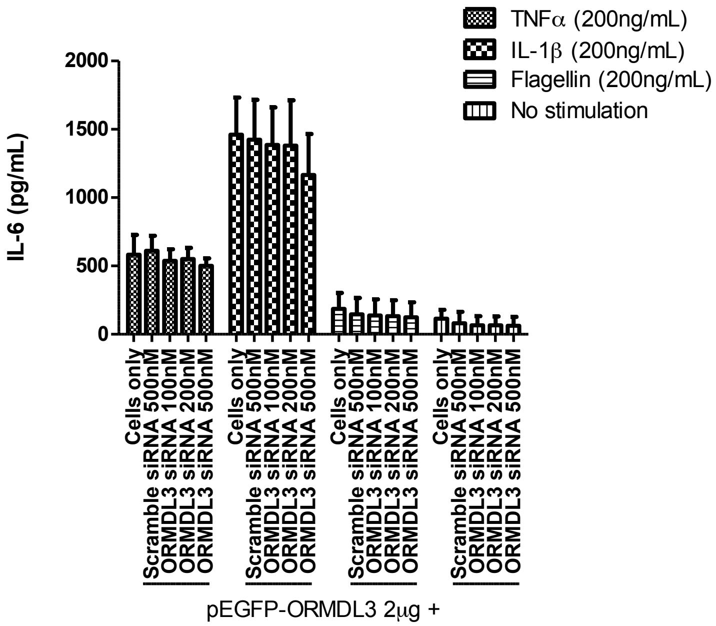

B

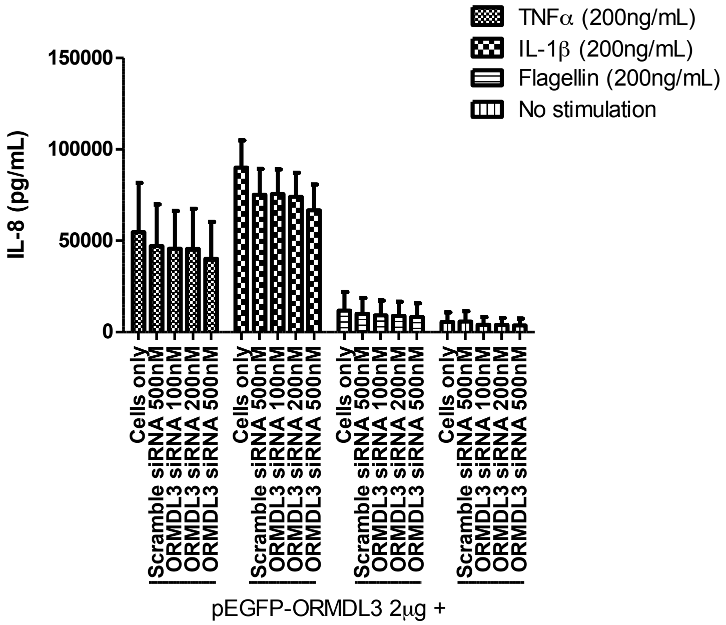

Cytokine production in plasmid and siRNA co-transfected A549 cells. A) Secreted IL-6 levels and B) secreted IL-8 levels after cell stimulation. Cells were stimulated with TNFα, flagellin, or IL-1β for 24 hours. Data represent the mean ± SEM of three experimental repeats. Statistical analysis was performed using one-way ANOVA with Bonferroni post-test.

A

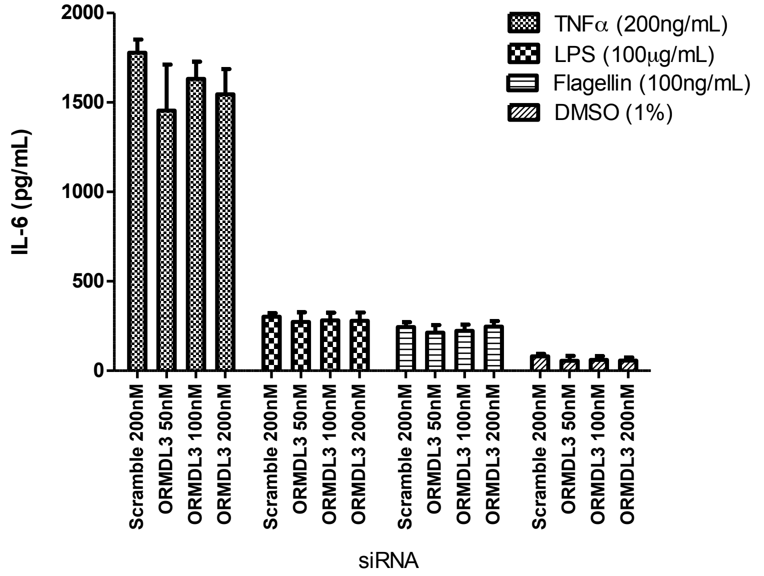

B

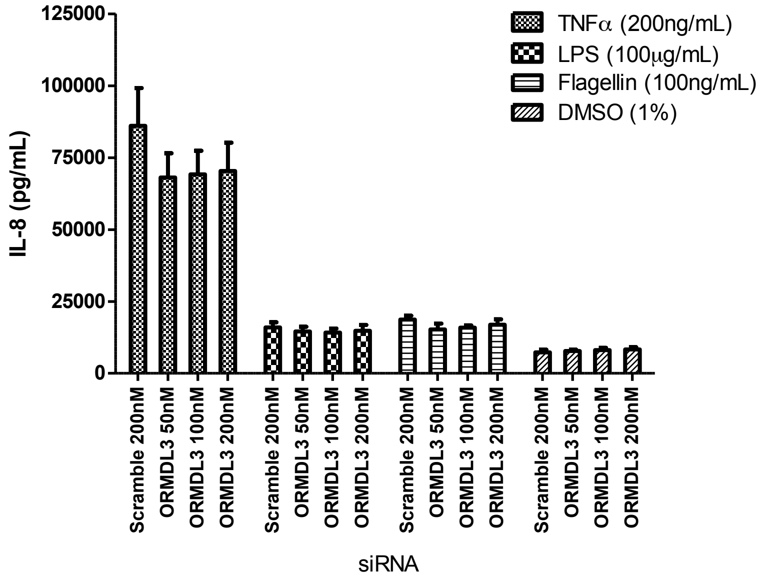

Cytokine production in siRNA-transfected 1HAE cells. A) Secreted IL-6 levels and B) secreted IL-8 levels after cell stimulation. Cells were stimulated with TNFα, LPS, flagellin, or DMSO (vehicle control) for 24 hours. Data represent the mean ± SEM of four experimental repeats. Statistical analysis was performed using one-way ANOVA with Bonferroni post-test.

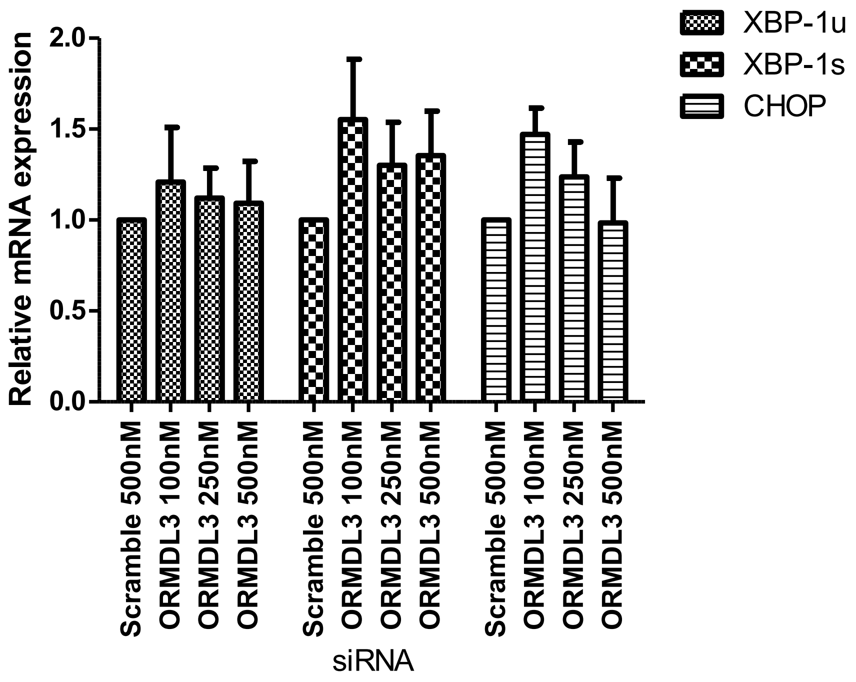

Expression of UPR gene markers in unstimulated A549 cells with *ORMDL3* knockdown. Three gene markers, *XBP-1u, XBP-1s,* and *CHOP*, were quantified 72 hours post-transfection. *XBP-1* is spliced upon activation of the IRE1 branch of the UPR and may lead to inflammatory responses. *CHOP* is induced through the PERK and ATF6 pathways and may lead to apoptosis. Data represent the mean ± SEM of three experimental repeats. Statistical analysis was performed using one-way ANOVA with Bonferroni post-test.

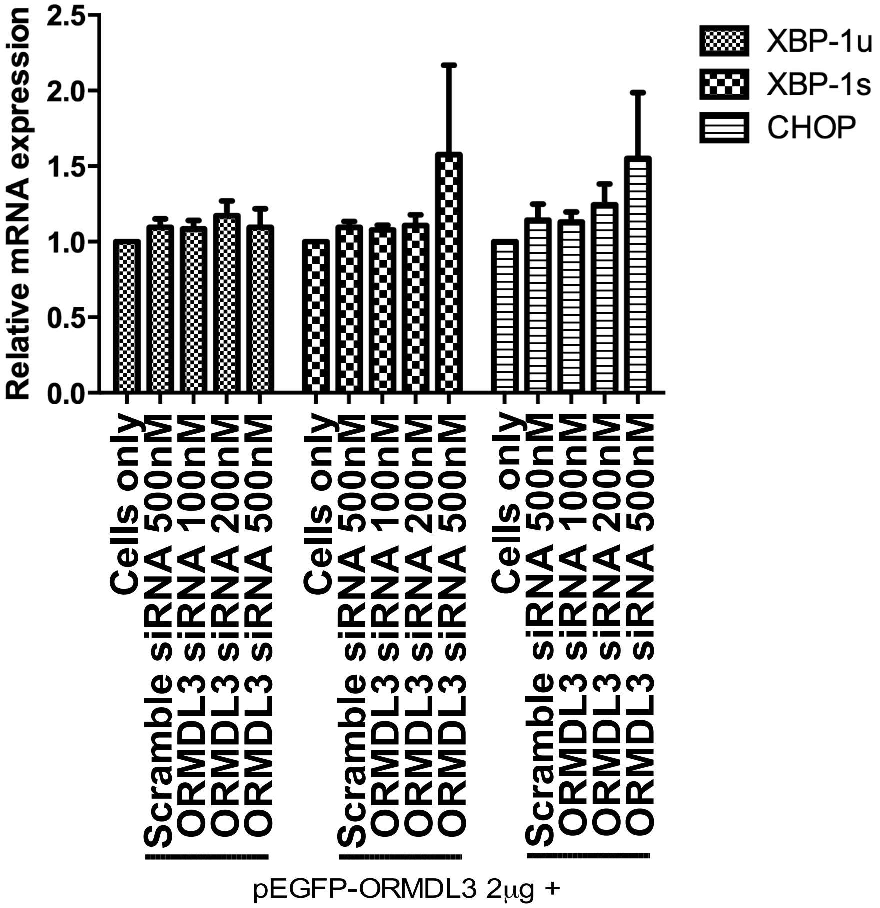

Expression of UPR gene markers in unstimulated 1HAE cells with *ORMDL3* knockdown. Three gene markers, *XBP-1u, XBP-1s,* and *CHOP*, were quantified 24 hours post-transfection. *XBP-1* is spliced upon activation of the IRE1 branch of the UPR and may lead to inflammatory responses. *CHOP* is induced through the PERK and ATF6 pathways and may lead to apoptosis. Data represent the mean ± SEM of three experimental repeats. Statistical analysis was performed using one-way ANOVA with Bonferroni post-test.
